# Behavioral Variation in Gorillas: Evidence of Potential Cultural Traits

**DOI:** 10.1371/journal.pone.0160483

**Published:** 2016-09-07

**Authors:** Martha M. Robbins, Chieko Ando, Katherine A. Fawcett, Cyril C. Grueter, Daniela Hedwig, Yuji Iwata, Jessica L. Lodwick, Shelly Masi, Roberta Salmi, Tara S. Stoinski, Angelique Todd, Veronica Vercellio, Juichi Yamagiwa

**Affiliations:** 1 Max Planck Institute for Evolutionary Anthropology, Leipzig, Germany; 2 Kyoto Prefectural University, Graduate School of Life and Environmental Sciences, Kyoto, Japan; 3 Dian Fossey Gorilla Fund International, Atlanta, GA, United States of America; 4 School of Anatomy, Physiology and Human Biology, The University of Western Australia, Crawley (Perth), WA 6009, Australia; 5 Interdepartmental Doctoral Program in Anthropological Sciences, Stony Brook University, Stony Brook, New York, United States of America; 6 Muséum National d¹Histoire Naturelle, Musée de l’Homme, National Center for Scientific Research (CNRS), University Paris Diderot (Paris 7) Éco-anthropologie et Ethnobiologie, Paris, France; 7 Department of Anthropology, University of Georgia, Athens, GA, United States of America; 8 World Wildlife Fund-CAR, Bangui, Central African Republic; 9 Kyoto University, Graduate School of Sciences, Kyoto, Japan; University of Florence, ITALY

## Abstract

The question of whether any species except humans exhibits culture has generated much debate, partially due to the difficulty of providing conclusive evidence from observational studies in the wild. A starting point for demonstrating the existence of culture that has been used for many species including chimpanzees and orangutans is to show that there is geographic variation in the occurrence of particular behavioral traits inferred to be a result of social learning and not ecological or genetic influences. Gorillas live in a wide variety of habitats across Africa and they exhibit flexibility in diet, behavior, and social structure. Here we apply the ‘method of exclusion’ to look for the presence/absence of behaviors that could be considered potential cultural traits in well-habituated groups from five study sites of the two species of gorillas. Of the 41 behaviors considered, 23 met the criteria of potential cultural traits, of which one was foraging related, nine were environment related, seven involved social interactions, five were gestures, and one was communication related. There was a strong positive correlation between behavioral dissimilarity and geographic distance among gorilla study sites. Roughly half of all variation in potential cultural traits was intraspecific differences (i.e. variability among sites within a species) and the other 50% of potential cultural traits were differences between western and eastern gorillas. Further research is needed to investigate if the occurrence of these traits is influenced by social learning. These findings emphasize the importance of investigating cultural traits in African apes and other species to shed light on the origin of human culture.

## Introduction

Culture in non-human animals, defined as ‘group-typical behavioral patterns shared by community members that to some degree are reliant on socially learned and transmitted information [1,2], has sparked much interest among scientists for several reasons, but particularly because of the implications for understanding the origins of culture in humans [2–4]. Cultural traits may originate from innovation, followed by diffusion among individuals through social learning (i.e. ‘learning as a result of interacting with or observing another individual or its products’[5]. Several approaches have been used to address the challenge of demonstrating the existence of cultural traits and social learning in wild animals [6]. Observational studies providing evidence that within- and between- population variation in behavior is a result of social learning and transmission have argued that there are social traditions or culture in many species including chimpanzees [7–9], bonobos [10], orangutans [11], capuchin monkeys [12,13], spider monkeys [14,15], meerkats [16,17], dolphins [18,19], whales [20] and birds and fish [21]. Additionally, experiments in captivity and the wild have shown that different methods of solving complex tasks can be transmitted among group members (e.g. [21–25].

Cultural traits in animals span the domains of diet [26,27], foraging techniques [8,16,28,29], tool use [7,30,31], and social interactions [13,14,32,33]. Cultural social interactions may include ‘social conventions’, which are defined as dyadic social behaviors or communicative behaviors which are unique to particular groups or cliques [13,34,35]. Behavioral traits considered to be cultural in non-human animals may result in improved nutritional intake, strengthened social bonds, or exhibit no obvious fitness advantage in some cases [12,36]. As a way to emphasize the complexity of cultural phenomena, some authors have restricted the presence of culture to those species with traditions in at least two different behavioral domains [35].

A commonly used starting point for demonstrating the existence of culture in wild animals is to use a geographic approach, or method of exclusion, in which researchers look for the presence/absence of particular behaviors in different social groups or populations (e. g. [9,11,13,14]). Following the method of exclusion, one can argue that a trait is cultural if a) a behavioral trait is customary (performed by most individuals of a particular age/sex class) or habitual (performed by several individuals of a particular age/sex class) in at least one site but absent in at least one site, b) if both ecological and genetic explanations can be inferred to be excluded as the explanation and c) if innovation and/or social learning can be inferred (ibid). This cross-site comparison approach has been criticized because conclusively eliminating any ecological or genetic influence on the behavioral variability is difficult, if not impossible, and because it relies on inference of social learning (e.g. [2], whereas some of the traits may be acquired through reinvention [37]). However, cultural traits are likely to be influenced by ecological conditions since ecology, genetics, and culture interact [38, 39].

To address the concern that ecological variation, not culture, may explain behavioral variation, detailed field studies in which behavioral variability was measured, while accounting for fine scale ecological variability, have provided more convincing evidence of cultural trait variation between populations [8] and between neighboring social units [7,17, 36]. Addressing whether or not there is a genetic influence on behavioral variability in wild populations is complicated. Langergraber et al. [40] found that levels of genetic and behavioral similarity were strongly correlated among chimpanzee populations and that only a few behaviors varied between genetically similar groups; yet these results do not eliminate the possibility that the traits are cultural because a) such a correlation does not mean that the occurrence of particular traits is solely genetically driven and causing the observed pattern of similarities and differences and b) some variation is still observed between genetically similar groups. Furthermore, according to the diffusion hypothesis, the spread (or diffusion) of a cultural trait is likely to radiate from its point of origin, such that behavioral similarity and geographic distance should be negatively correlated [11]. Geographic distance among populations is likely to be negatively correlated also to genetic similarity, making it difficult to disentangle the effects of social diffusion and genetic influence. Furthermore, the degree that a trait is acquired by individuals (via mechanisms such as stimulus enhancement or emulation learning or individual reinvention) may vary depending on the difficulty of the trait in relation to the ‘zone of latent solutions’ of the species [37]. Nonetheless, the method of exclusion is a useful approach for cataloguing behavioral variation of potential cultural traits for further study of causes of variation (hidden environmental and/or genetic), underlying mechanisms of social learning, and evidence of cultural variation [41].

**Fig 1:**
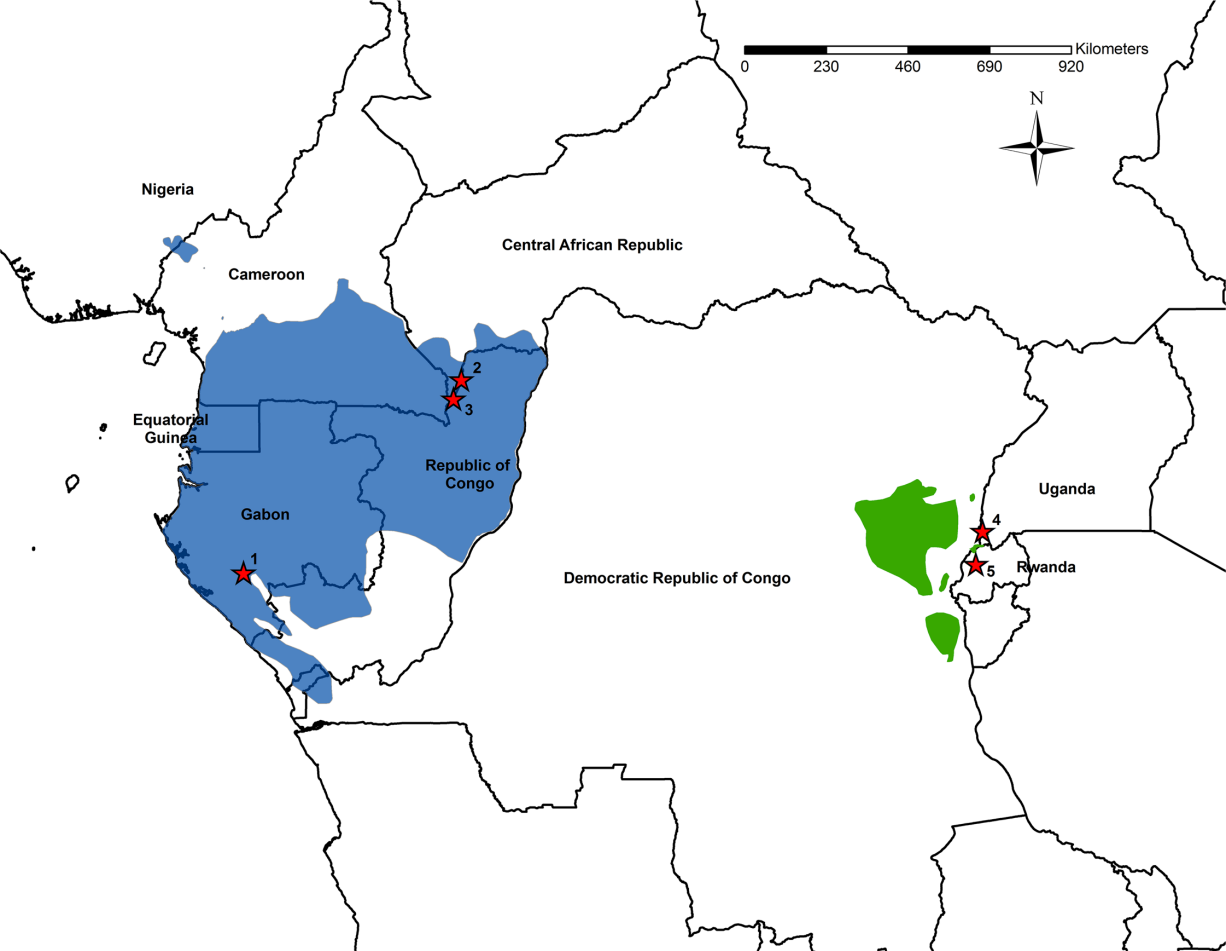
Map of the five gorilla study sites. Western Gorillas: 1. Moukalaba-Doudou National Park, Gabon 2. Bai Hokou, Dzanga-Ndoki National Park, Central African Republic 3. Mondika, located along the border of Dzanga-Sangha National Park, Central African Republic and Nouabale Ndoki National Park, Republic of Congo. Mountain Gorillas: 4. Bwindi Impenetrable National Park, Uganda 5. Karisoke Research Center, Volcanos National Park, Rwanda. Blue indicates the distribution of western gorillas (*Gorilla gorilla*) and green indicates the distribution of eastern gorillas (*Gorilla beringei*).

Among the great apes, the least amount of evidence for culture and social learning is available for gorillas [42,43]. However, gorillas possess characteristics that are predicted to promote social learning and transmission such as a long developmental period, overlapping generations, and regular social interactions among group members living in stable social groups [44]. Gorillas live in a wide variety of habitats and they exhibit variability in morphology, diet, behavior, and social structure [45–49]. The genus *Gorilla* was considered one species until 2001 when it was reclassified to be divided into two species, each having two subspecies [50]. Western gorillas (*Gorilla gorilla*) are found in seven central African countries and eastern gorillas (*Gorilla beringei)* are found in three, with the two species being separated by approximately 1000 km (Fig 1). The two species are believed to have initially split roughly 1.2–3 MYA, but with some gene flow until as recently as 80,000–200,000 years ago [51–53]. Each species consists of two subspecies and this study examines only the subspecies *Gorilla gorilla gorilla* (western gorillas) and *Gorilla beringei beringei* (mountain gorillas).

Despite large variation in ecological conditions, the basic social structure of gorilla groups is consistent across all localities, with groups composed of an average of ten individuals with at least one adult male (silverback), several adult females and offspring [46,54]. However, group size may vary considerably, with groups containing more than one silverback and more than 20 individuals occurring almost exclusively in mountain gorillas [47]. Even after nearly five decades of research, the majority of our knowledge about gorillas comes from the Karisoke Research Center in Rwanda, which is located at an ecological extreme for primates with some groups living at over 3000m altitude. Our limited knowledge of other gorilla populations until recently stemmed largely from the difficulties of habituating western gorillas and hence an inability to collect detailed data on their behavioral patterns, and only limited efforts to study other populations of eastern gorillas. However, in the past decade additional study sites of habituated gorillas have developed, providing an opportunity to examine variability in their behavior.

The first goal of this paper is to generate a list of potential cultural traits in wild gorillas from five sites by examining variation in the occurrence of behavioral traits that could potentially be influenced by social learning and not due to ecological or genetic variation (using the method of exclusion). We then classify these traits according to behavioral domains (e.g. foraging, environment related, social, or gestural). Second, we quantify the degree of dissimilarity across sites and examine the relationship between geographic distance and behavioral dissimilarity. We expect behavioral dissimilarity to increase as the distance between study sites increases. Lastly, we discuss rarely observed behaviors that may reflect innovations [55] as well as behaviors that were present in all sites but varied in frequency of occurrence, as they may be potential universal cultural traits [15].

## Methods

### Ethics Statement

This study was conducted with wild free-ranging animals and was completely observational. Research was conducted in accordance with guidelines of the national authorities where the work occurred. Approval and permission to conduct research was granted by the Uganda Wildlife Authority, the Uganda National Council of Science and Technology, the Rwanda Development Board, Ministry of Education and Water and Forests of the government of Central African Republic for permission to work in Dzanga-Ndoki National Park, the Ministries of Scientific Research and Forest and Water in the Central African Republic and Republic of Congo for permission to conduct research at Mondika, and the Centre National de la Recherche Scientifique (CENAREST) and the Agence Nationale des Parcs Nationaux (ANPN) in Gabon.

### Study Sites

Data were collected from five sites with habituated gorillas (Fig 1; Table 1). Gorilla groups were followed on a near daily basis at all sites, observed for a minimum of four hours per day, and observations of focal animals were conducted on a regular basis on various topics (i.e. diet, social interactions, communication [56–66]). The three western gorilla sites are separated from the two mountain gorilla sites by approximately 1500–2100 km (see Table 2 for distance among all study sites). All the groups were monitored on a nearly daily by field assistants and we report the observation time (days) by the people contributing to this project (Table 1).

**Table 1:** Details of the observations made at the five study sites. Total group size and the age sex classes include the range of number of individuals during the course of the study. Not all groups were observed for the entire duration of the study period for the sites with more than one group.

Study Site	Subspecies	Annual Rainfall (mm)	Geographic Coordinates	Altitude (for study groups)	Number of Observers	Days of Observation	Observation Period	Number of Groups	Group Name	Total Size (range during study period)	# Silverback	# Blackback	# Adult Female	# Juvenile & Subadult	# Infant
Bwindi	*G*. *bberingei*	1180	1° 0' S 29° 40' E	2100–2500 m	2	957	1998–2011	1	Kyagurilo	9–21	1–4	0–4	5–7	0–6	0–7
Karisoke	*G*. *b*. *beringei*	2050	1° 50' S 29° 30' E	2700–4000 m	2	852	2006–2014	11	BEE	21–27	4–7	0–2	7–10	7–13	+
	* *								INS	2–8	1	0	1–4	0–4	+
	* *								ISA	4–17	1–2	0–1	2–8	0–8	+
	* *								KUY	8–16	2–3	0–2	2–6	2–11	+
	* *								NTA	5–19	2–4	0–2	1–7	0–9	+
	* *								PAB	29–65	3–8	1–10	7–20	11–35	+
	* *								SHI	23–29	4–7	1–2	6–11	7–14	+
	* *								TIT	4–14	1–4	0–3	1–4	0–8	+
	* *								UGE	6–23	1–3	0	1–9	1–14	+
	* *								URU	4–7	1	0	2–3	1–4	+
	* *								BWE	2–12	1	0	1–7	0–8	+
Bai Hokou	*G*. *g*. *gorilla*	1600–1800	2° 50' N 16° 28' E	340–615 m	3	2348	2004–2014	3	Munye	3	1	0	1	1	0
									Makumba	9–13	1	1	2–4	3–7	5
									Mayele	14–16	1	0	2–4	8	4
Mondika	*G*. *g*. *gorilla*	1600–1800	2° 21' N 16° 16' E	<400m	3	815* (and 47 months)	2003–2010	3	Kingo	9–13	1	0	4–6	1–2	3–5
	* *								Muya	3	1	0	2	0	0
	* *								Buka	13	1	1	4	3–4	3
Moukalaba	*G*. *g*. *gorilla*	1300–1800	2° 3' S 10° 57' E	<200 m	3	430	2007–2015	1	Gentil	19–23	1	0–5	5–9	5–11	3–7

* another researcher made observations at Mondika for a total of 47 months (but exact number of days is not known). + for the Karisoke study groups, infants are combined in the juvenile and subadult age category.

**Table 2:** Overall behavioral dissimilarity between gorilla study sites using Manhattan distances (above diagonal; see methods for calculation) and geographic distance (below diagonal) in the ten pairs of study sites.

	Bwindi	Karisoke	Mondika	Bai Hokou	Moukalaba
Bwindi		25	41	42	44
Karisoke	45		33	42	48
Mondika	1514	1517		19	25
Bai Hokou	1509	1513	60		16
Moukalaba	2095	2081	780	810	

Three groups of western gorillas (*Gorilla gorilla gorilla*) were observed at Bai Hokou, Dzanga-Ndoki National Park, in south-western Central African Republic (CAR), three groups were observed at the Mondika Research Center which straddles the border of the Central African Republic and the Republic of Congo, and one group was observed in Moukalaba-Doudou National Park, Gabon. Bai Hokou and Mondika are separated by only 60 km of contiguous rainforest. Both are composed primarily of low altitude (<400 m) mixed-species semi-evergreen forest [67,68]. The habitat of the gorillas in Bai Hokou includes open clearings, referred to as ‘bais’, whereas part of the home range of the groups in Mondika is swamp. Moukalaba-Doudou is located approximately 800 km away from those two sites and is composed of secondary forest, *Musanga cecropioides* forest, and savanna [69]. Observations of habituated gorillas were made by three observers at each of these sites spanning (not concurrently) a time period of over four years. All groups of western gorillas included in the study contained only one silverback, whereas both one-male and multimale mountain groups were observed.

The two populations of mountain gorillas (*Gorilla beringei beringei*) are located approximately 30 km apart from each other at the nearest point (study groups approximately 45 km apart), separated by an expanse of cultivated and developed land, preventing any migration between the two areas for at least the past several hundred years [70]. Three groups of mountain gorillas at the Karisoke Research Center, in the Volcanoes National Park, Rwanda were observed regularly for a period of three years (2006–2008) and following various group fissions and new group formations, an additional six groups were observed through 2014. The Virunga Volcanoes is the only location inhabited by gorillas that does not contain large fruiting trees. One group of gorillas was observed for 13 years in Bwindi Impenetrable National Park, Uganda.

### Survey Procedure

First, based on our knowledge of gorilla behavior we generated a list of 41 behaviors that were likely to vary across sites. For each site, we categorized each behavior following [11], which is slightly modified from [9]. Numerical codes were assigned to each behavioral category following [40]. The categories and numerical codes used were as follows:

Customary (3): when the behavior occurred in all or most members of at least one age-sex class.

Habitual (2): when the behavior was observed repeatedly in several individuals, but was not customary.

Present (1): when the behavior was observed, but was neither customary nor habitual.

Absent (0): when the behavior was not observed and no ecological explanation is apparent.

Ecological Explanation: when the behavior was absent because of a local ecological feature, such as the presence or absence of a particular plant.

We then examined the results across sites and classified each trait as either variable across sites or universal among all sites. Finally, we were left with traits that varied among sites, excluding those with a straightforward ecological explanation. We considered traits that were classified as either customary or habitual in at least one population and absent in at least one other as potential cultural traits following Whiten et al. [9] and van Schaik et al. [11]. Behaviors that were neither customary nor habitual at any site (e.g. only present at one or more site) were not included as potential cultural variants (following previous studies) but are reported here as rare behaviors. We also categorized the traits as being foraging techniques, gestures, a component of social interactions among individuals, or environment related (those that involved a physical component of the environment and not another gorilla, but were not related to foraging). We next sorted potential cultural traits based on whether they differed within species, between species (i.e. western and eastern gorillas), or with no apparent pattern. Traits that were excluded for ecological explanations were those that could not occur at some sites due to ecological conditions, but occurred in all locations with suitable ecological conditions.

To test the prediction that behavioral dissimilarity increases as the distance between study sites increases, we used the numerical codes of the traits that were deemed as potential cultural variants to calculate the overall behavioral dissimilarity among the ten dyads of gorilla sites. Following Langergraber et al. [40], we used the Manhattan distance, which is the sum of the absolute values of the differences between the behavioral variants for a dyad of study sites (i.e. if the numerical code for a trait at one site is three, but one at another site is one, the absolute difference is two). Next, differences for all behavioral variants per dyad were then summed. The greater the difference between two study sites, the larger the difference in behavioral patterns. Because subtraction requires two numbers, behavioral variants with missing values (i.e. due to ecological explanations; for example, termites not present in mountain gorilla habitat) were excluded pairwise but not from the entire list. This does not bias the results because we are interested in the amount of behavioral dissimilarity that is due to potential cultural variation. We compared the behavioral dissimilarity among populations with geographic distance between locations using Pearson correlation coefficients calculated with a Mantel matrix permutation using 10,000 permutations to account for each population occurring multiple times in all the comparisons (ten dyadic comparisons among the five study sites).

## Results

From the 41 behaviors examined, we identified 23 potential cultural traits (Table 3), which were habitual or customary at least at one site while absent from at least one other site. Of these, one was foraging related, nine were environment related, seven involved social interactions, five were gestures, and one was communication related. Roughly half (52%) of the potential cultural traits was variable within a species, whereas the other half (48^) distinguished the western from mountain gorillas. For the twelve traits that did not show clear differences between species, absence was recorded in at least one population and there were varying degrees of occurrence among the other populations with no clear pattern between the western and mountain gorillas. Comparing between the two mountain gorilla sites, of the 23 potential cultural traits, seven were absent at one site but occurred in the other, six were absent at both, eight occurred in both study sites, and two had ecological explanations for the absence at one location. Comparing among only the three western gorilla sites, ten behaviors were absent at one or two sites, four were absent at all three sites, and nine occurred at all three sites.

**Table 3:** Variation in behavioral traits among five gorilla sites.

		Mountain Gorillas		Western Gorillas		
Behavior	Behavioral Domain	Karisoke	Bwindi	Moukabala	Bai Hokou	Mondika
**Potential Cultural Traits with Variation among species & sites; absent in at least one site**						
Using teeth as '5th limb' while climbing trees	E	absent	customary	present	absent	present
Staring at reflection in water (as if looking into a mirror)	E	habitual	absent	absent	present	present
Play-rolling downhill	E	customary	present	absent	habitual	absent
Sitting in water 'basin'	E	absent	absent	customary	absent	absent
Lick water off of arm after it rains	E	absent	present	habitual	absent	absent
Putting both arms on other individuals’ back while moving/traveling, sometimes several individuals in a row (not as part of play)	S	customary	absent	habitual	present	customary
Play-chase each other around a tree	S	customary	absent	habitual	present	present
Immatures playing *on* the silverback	S	customary	customary	present	absent	customary
Immatures playing *with* the silverback	S	customary	present	absent	present	customary
Tree slap–use hands to beat against a tree, in the same manner as a chest beat	G	absent	customary	customary	customary	present
Tapping head with hand	G	customary	present	absent	absent	absent
Pseudo-feeding (putting a food or non-food plant in mouth, with a part of it hanging out, without ingesting the item); as part of a display	G	customary	absent	customary	customary	customary
**Potential Cultural Traits with Variation between mountain and western gorillas**						
Cleaning fruit–rubbing fruit against arm or body, presumably to remove dirt; for some fruit it may be to remove spines (e.g. some *Diosporus* sp)	F	ecological	absent	customary	customary	customary
Bridge-making–break or bend branches and place over water on the edge of stream or swamp and then walk across it to avoid getting wet.	E	absent	absent	absent	present	customary
Bare earth nest–nest on the ground without breaking or bending any vegetation.	E	absent	absent	customary	customary	customary
Cup hands, fill with water and drink (eg. not drinking directly from source with mouth)	E	absent	absent	customary	habitual	present
Bipedal walking/wading across water	E	absent	absent	customary	customary	present
Females embracing silverback in reaction to a male display, seemingly appeasement behavior, with or without giving ‘grumble’ vocalization	S	customary	customary	absent	absent	absent
Silverback-Adult Female Grooming	S	customary	customary	present	absent	absent
Adult Female-Adult Female Grooming	S	customary	customary	absent	absent	absent
Hand clapping	G	absent	absent	customary	customary	customary
Splash displays (displaying through water in bai or stream)	G	absent	absent	habitual	habitual	absent
Blowing raspberries–pursing lips and blowing air through, to produce a ‘farting’ sound vocalization. (also described for orangutans)	C	habitual	present	absent	absent	absent
**Universals: Variation in behavioral traits among sites, but present at all sites**						
Day nest–adult makes a nest & uses it during the day	E	customary	present (rare)	customary	present (rare)	customary
Nest site reuse	E	present	rare	customary	present	rare
Immatures carrying infants–dorsally	S	habitual	present (rare)	customary	customary	customary
**Rare Behaviors; absent at most sites**						
Thistle processing—'rolling method'	F	absent	present	ecological	ecological	ecological
Nest reuse (reusing actual nest)	E	absent	absent	present	absent	absent
Mouth-washing: taking water into mouth, then moving it back and forth within mouth before swallowing.	E	absent	absent	absent	present	absent
Dig a hole on edge of stream or bai with hand, wait for it to fill with water and then drink it.	E	absent	absent	absent	present	absent
Tooth brushing: rubbing fingers against teeth	E	absent	absent	absent	present	absent
Covering lap with vegetation during resting	E	present	absent	absent	absent	absent
Shaking young tree leaves to clean off dirt	E	absent	absent	present	absent	absent
Dipping arm into water and using it as a sponge	E	present	absent	present	present	absent
Returning to dead individuals ('mourning')	S	present	present	absent	present	insufficent data
‘Rain dance’–chest-beating and displaying as it starts to rain; specify age/sex class	G	absent	absent	absent	present	present
**Traits with Ecological explanation for presence or absence at some sites**						
Washing swamp foods–wash the mud off the roots of Hydrocharis	F	ecological	ecological	ecological	habitual	customary
Soil scratching–specific to *Gilberteodendron* forest? (i.e. eating truffles at Bai Hokou)	F	ecological	ecological	present	customary	customary
Termite feeding: ‘pound-on-hand’ technique	F	ecological	ecological	customary	customary	customary
Termite feeding: ‘remove-with-tongue’ technique	F	ecological	ecological	customary	customary	customary
Thistle processing–using basic method	F	customary	customary	ecological	ecological	ecological

Type of Behavioral Domain: F = foraging; E = environment related; S = social context; G = gesture, C = communication.

The remaining 18 traits could not be considered as potential cultural traits (Table 3). Three traits were observed in all populations but at varying degree of occurrence, so could be considered as universals. Five could be eliminated due to ecological explanations, with the trait occurring at all locations with suitable ecological conditions. Lastly, ten behaviors were rarely observed in one or more populations and were not observed to the level of habitual in any population.

**Fig 2:**
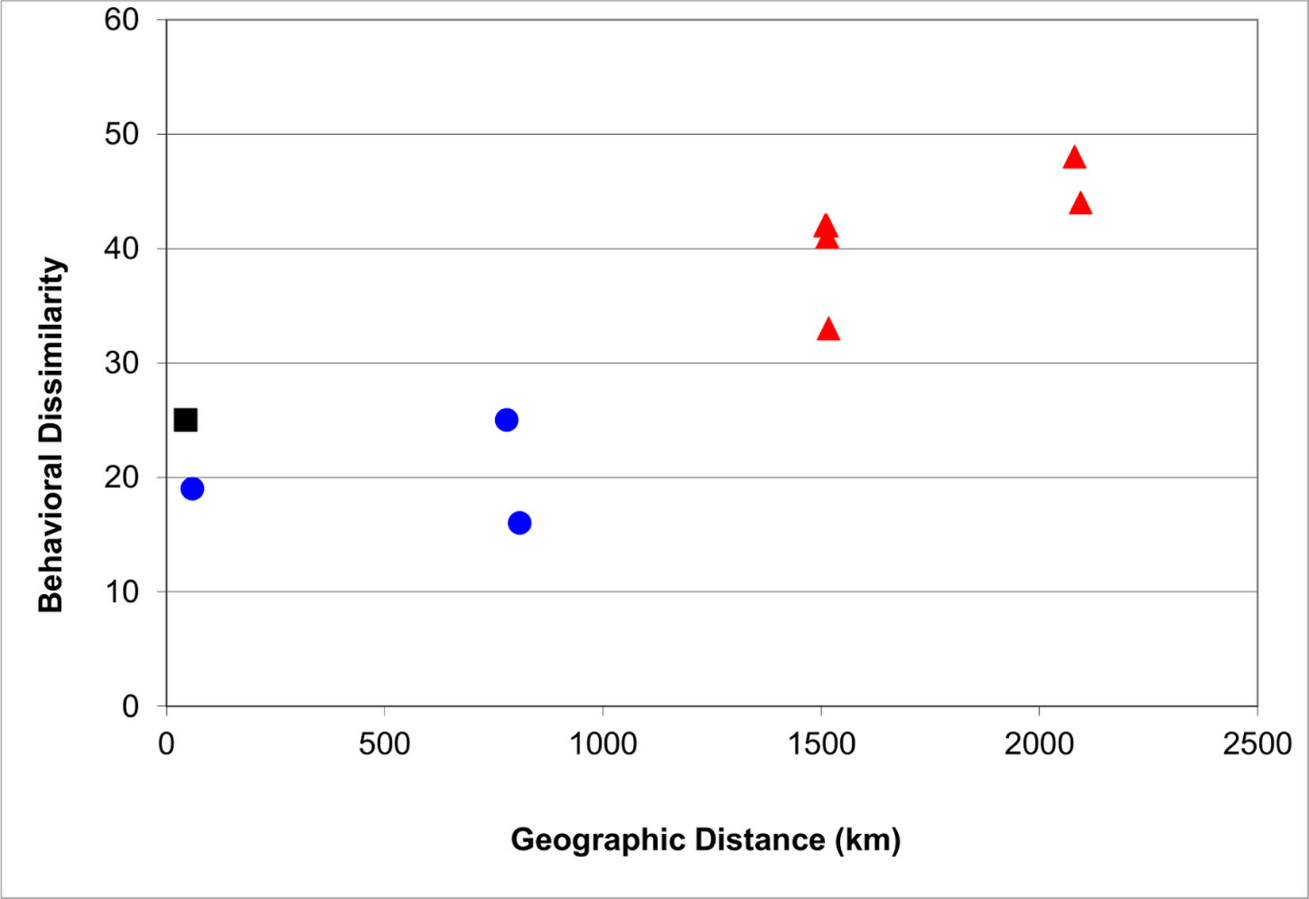
Behavioral dissimilarity (quantified as Manhattan distance; see methods for explanation) plotted against geographic distance among five gorilla study sties. Each data point represents a dyad of two sites (two of the 10 dyads have the same value; hence only nine points are visible). As the geographic distance between sites increases, the dissimilarity in behavioral traits increases.

The behavioral dissimilarities, quantified using the Manhattan distances, ranged between 16 and 48 (n = 10; Table 2; the highest possible score was 69). The four lowest Manhattan distances (having most behavioral similarity) were in the dyad containing the two mountain gorilla populations and dyads where both populations were western gorillas (Table 2). Geographic distances among the sites ranged from 45 to 2095 km (Table 2). The behavioral dissimilarities and geographic distance between sites were strongly correlated (Pearson’s r = 0.930; p = 0.008; Fig 2).

## Discussion

This study adds to the growing body of evidence of behavioral variants across many taxa that may be considered as potential cultural traits. We observed variation in occurrence of behaviors among gorillas at five field sites spanning behavioral domains of social interactions, gestural communication, environment related behaviors, and one foraging behavior. The strong correlation between the behavioral dissimilarity and geographic distance (a proxy for genetic similarity) indicates that a genetic influence cannot be ruled out as affecting the occurrence of the behavioral traits among the populations, particularly between the mountain gorillas and western gorillas, but it does not exclude the possibility of social learning. Alternatively, the correlation between the behavioral dissimilarity and geographic distance provides support for the behavioral diffusion hypothesis, in which there is more similarity in behavioral patterns among nearby populations and declines as distance increases because the trait has not spread further. However the low score between two western gorilla sites that are far from each other (Bai Hokou and Moukalaba), indicating that they have high similarity in the occurrence of traits, in comparison to Bai Hokou and Mondika, which are only 60 km apart from each other, would argue against both the genetic and diffusion hypotheses. Social learning and transmission may occur through a variety of processes including stimulus enhancement and emulation (see Whiten [71] for review). It is also possible that these traits were spontaneously reinvented at the different sites based on the ‘zone of latent solutions’ hypothesis [37].

We observed variation in the occurrence of half of the potential cultural traits between the two mountain gorilla sites, among the three western gorilla sites, and among all five sites (Table 3). Half of the behavioral variants reflected differences between western gorillas and mountain gorillas, which currently are classified as subspecies of two different species. We believe making a comparison on the species level is valid, as a similar approach was taken when examining culture in two species of orangutans [11,72], which diverged genetically 1.5 million years ago. Additionally, a large proportion of cultural variants observed in chimpanzees occur only in the most genetically divergent of the four subspecies [40,73], the West African *Pan troglodytes verus*, which has been proposed as a different species [74]. These examples emphasize the difficulty in observational studies of completely removing the possibility of genetic influences on the occurrence of traits [40,75]. Nonetheless, our results are consistent with evidence of potential cultural traits in both species of gorillas.

Only one trait involved food processing (rubbing fruit on arm to clean it) and nine of the 23 (39%) potential cultural traits were environment related. This diverges from studies of both chimpanzees and orangutans in which the majority of traits involved object manipulation and/or food processing [9,11,73]. In contrast to other ape species, potential tool use by wild gorillas has been observed only very rarely [58,76,77]. The question of why tool use in wild gorillas is so rare remains intriguing, especially given its frequency in captive gorillas. Wild bonobos also rarely use tools, which may be due to having less intrinsic motivation to manipulate objects compared to chimpanzees, rather than resulting from ecological conditions [78].

However, food processing techniques for several plants species consumed by mountain gorillas and termites eaten by western gorillas are seemingly complex and can be achieved through different methods [79]. The coarse level of defining these behaviors (termite eating and thistle processing) in this study resulted in no variation among sites where these behaviors occurred. Detailed, quantitative field studies on the variation among individuals and ontogeny of these behaviors are rare, but these traits are likely candidates for social learning [79], but see [80]. Two methods of termite eating have been observed in western gorillas, but both techniques were observed at the three sites in this study that have termites so it remains unclear if such techniques were individually acquired, reinvented, or socially learned. Similarly, the two methods of extractive foraging used by banded mongoose have been shown to be socially learned, even though most individuals use both methods [24]; see also [81] for extractive foraging by capuchin monkeys), stressing the importance of conducting social learning studies because the method of exclusion tends to conservatively eliminate traits for which multiple variants are found in all populations. Furthermore, research is needed to exclude the possibility that variation in environment related traits (e.g. bridge making, bare earth nests) are not a result of variation in environmental conditions (e.g. depth of water, mean temperature) on a finer scale than what was considered in this study (see [8,82]).

The social interactions, the gestures, and the communication related trait that varied in usage among the gorilla populations (13 of 23 traits) are difficult to exclude as cultural traits because they do not rely on particular components of the environment. Nonetheless, ecological variation could also play a role in the occurrence and frequency of social interactions. Social conventions, or variation in the occurrence of social behaviors, may be the result of differences in socialization during maturation or in the context of the social environment [33,81]. Of particular interest, grooming among adult females in wild western gorillas was never observed and grooming among adult females and a silverback was observed at only one site; although mother-offspring grooming was observed regularly at all sites. Given the ubiquity of grooming among primates, including both related and unrelated dyads of mountain gorillas [83], and its function in maintaining social relationships [84], this is a unique omission to the behavioral repertoire of wild western gorillas [85], or at least in these groups. Grooming among adult western gorillas in captivity has only rarely been mentioned in the published literature [86]. Observations of additional wild western gorillas are necessary to determine if this is a species-wide phenomena or if grooming is a social behavior that has gone extinct in these locations (for more on extinction of social conventions see [13,87].

Several gestures and social traits were observed in one population of mountain gorillas but not the other (e.g. play chase around tree, arms on back of gorilla in front while travelling, pseudo-feeding as part of display) or were not observed in all three western gorilla populations (silverback-adult female grooming, splash displays, immatures play on or with the silverback), which perhaps offer some of the strongest support for behavioral variants being cultural since these traits are by nature social (less likely to be environmentally influenced) and some of these populations (Bwindi and the Virungas; 70) have been isolated from one another only relatively recently. Hand clapping, which was observed in western but not mountain gorillas, has also been recorded regularly in captive western gorillas [88,89]. Genty et al.[89] suggest that a large majority of gorilla gestures are part of a species typical repertoire, but that their use may be based on contextual learning because they are used in a highly flexible manner; this could include the lack of using some gestures in some locations. This interpretation of gestural communication does not preclude the possibility that their *use* can be socially learned and transmitted and therefore be considered cultural rather than ecologically or genetically driven.

As observed in the great apes, variation in the propensity to use cultural traits in different behavioral domains (e.g. foraging techniques, tool use, and social context) is also apparent among New World monkeys and most likely reflects a combination of morphological, ecological, and social adaptations. Foraging behavior has been one focus of traditions in white-faced capuchins [29], although they only rarely use tools [12,29]. In contrast, a wide diversity of tool use has been observed in brown capuchins [31,90]. Social conventions occur regularly in white-faced capuchins, but not brown capuchins [12]. Spider monkeys are morphologically limited in their ability to manipulate objects which may explain their lack of material culture, and most of the traditions occurred within a social context [14]. The relative use of social learning for ecological and social traits among the great apes may reflect adaptive strategies to cope with different ecological and social constraints, resulting in different evolutionary cognitive pathways and niche construction [91,92].

Following other studies [9,11], we have listed a handful of behaviors that were observed rarely, because they could be potential innovations [72,87,93]. For example, thistle rolling was observed repeatedly by only one subadult female gorilla at Bwindi [94], but unfortunately she dispersed into an unhabituated group a few months after she started performing this behavior so we were unable to observe if it was passed on to her offspring. The behavior ‘covering lap with vegetation during rest’ was observed only four times in one group of Karisoke mountain gorillas. This group shifted their range to a very high altitude (>3300 m) in recent years so the occurrence of the behavior may be an adaptation to ecological conditions and may spread to more group members.

In contrast to behaviors observed rarely in only one population, several behavioral traits were observed in all populations, but with differing degrees of occurrence. By definition, the method of exclusion disregards such universal behaviors (those that are present in all populations), but such behaviors may still be the result of innovation and social learning [73]. Several cases of group- or community-preferred behavioral variants have been documented spanning a variety of social and foraging behaviors [15,27,33,81]. Examining the occurrence of universal traits using systematic data collection methods that estimate frequencies, rates of behavior and statistical comparisons may broaden the repertoire of cultural traits in gorillas, if reinforced with evidence of social learning.

To better understand the potential cultural variants among gorillas, future research should focus on detailed systematic inter-individual comparisons of behavioral patterns within field sites or at ecologically similar sites [15, 72, 95] to reduce the possibility of ecological or genetic influences on traits, help to avoid the possibility of false absences, and to provide a more quantitative assessment of frequency and distribution of behaviors among group members. Focusing on possible modes of social transmission can help confirm the most likely method of trait acquisition (e.g. [81, 96]). Conducting studies on gorillas at ecologically similar sites can be challenging because of the large ecological variation within and among gorilla habitats and the small number of habituated groups. For example, there is large variation in plant species composition and density between and within the habitats of the only two populations of mountain gorillas [97, 98]. Western gorillas are found across a much larger range and ongoing habituation efforts should make more comparisons possible in the future, but ecological variation between sites may remain a limitation in some cases given the differences in habitat and diet (e.g.[99, 100]). This study serves as a starting point for adding gorillas to the discussion of how animals may exhibit behavioral variation acquired through social learning as an additional mode of adapting to changing social and ecological landscapes [101].
